# The Variety of 3D Breast Cancer Models for the Study of Tumor Physiology and Drug Screening

**DOI:** 10.3390/ijms24087116

**Published:** 2023-04-12

**Authors:** Eleonore Fröhlich

**Affiliations:** 1 Center for Medical Research, Medical University of Graz, 8010 Graz, Austria; eleonore.froehlich@medunigraz.at; Tel.: +43-316-3857-3011; 2 Research Center Pharmaceutical Engineering GmbH, 8010 Graz, Austria

**Keywords:** 3D models, breast cancer, spheroids, organoids, microfluidic techniques, bioprinting, tumor microenvironment

## Abstract

Breast cancer is the most common cancer in women and responsible for multiple deaths worldwide. 3D cancer models enable a better representation of tumor physiology than the conventional 2D cultures. This review summarizes the important components of physiologically relevant 3D models and describes the spectrum of 3D breast cancer models, e.g., spheroids, organoids, breast cancer on a chip and bioprinted tissues. The generation of spheroids is relatively standardized and easy to perform. Microfluidic systems allow control over the environment and the inclusion of sensors and can be combined with spheroids or bioprinted models. The strength of bioprinting relies on the spatial control of the cells and the modulation of the extracellular matrix. Except for the predominant use of breast cancer cell lines, the models differ in stromal cell composition, matrices and fluid flow. Organoids are most appropriate for personalized treatment, but all technologies can mimic most aspects of breast cancer physiology. Fetal bovine serum as a culture supplement and Matrigel as a scaffold limit the reproducibility and standardization of the listed 3D models. The integration of adipocytes is needed because they possess an important role in breast cancer.

## 1. Introduction

Breast cancer (BC) belongs to the common cancers and represented 11.7% of all new cancer cases and 6.9% of all deaths in 2020 in the whole world (https://gco.iarc.fr/today/data/factsheets/cancers/20-Breast-fact-sheet.pdf (accessed on 21 December 2022)). Breast cancer was also the most commonly diagnosed cancer type (12.5%) in 2021, accounting for one in eight cancer diagnoses worldwide [1]. Mortality rates decreased between 1989 and 2010 by 1.45–3.45% per year for all age groups (20–79 years), and between 2010 and 2017 by 1.2–2.2% per year in women aged 40–79 years and by 0.5% per year in the group aged 20–39 years in the US [2]. The decline was mainly due to early diagnosis, but better treatment options also contributed to this positive trend. Mortality from BC worldwide, however, is 16% [1], and improved diagnosis and treatments are needed.

Animal models are still the gold standard in studies on tumor physiology and for the validation of promising compounds for cancer treatment. However, the vast majority of drugs (90%), which successfully passed preclinical testing, failed in the clinical phases. The failure rate was 85% in the early clinical trials, but also only half of those that survived until phase III became approved for clinical use [3]. The largest proportion of failures occurred in trials for cancer drugs. The main reasons were lack of clinical efficacy (40–50%), unmanageable toxicity (30%), poor drug-like properties (10–15%), and lack of commercial needs and poor strategic planning (10%) [4]. Animal models in cancer overestimate by ~30% the likelihood for effective treatment. The impression of efficacy may also be distorted because many negative results are often not published [3]. Only around one third of the highly cited animal studies were verified in clinical trials and as little as 8% passed phase I successfully.

The problem of interspecies differences can be illustrated in non-human primates. Even if there is a difference in DNA of only 1–2% from humans, chimpanzees are not optimal models because the small difference results in ~20% of differences in protein between the species [5]. It is therefore not surprising that in an evaluation of 108 anti-tumor agents, the positive predictive value of toxic effects obtained in preclinical studies with mouse, rat, dog and monkey was 0.65, and the negative predictive value was 0.5 for human toxicity [6]. Other factors, such as a lack of best practice standards, laboratory environment (stress due to cage housing) and the use of only one sex of animals may also contribute to the poor predictive value of animal studies. It cannot be hoped that the success of therapeutic cancer vaccines is better than for small molecules because out of 23 phase II/III clinical trials, 18 failed [3]. Conventional 2D monocultures are insufficient to assess the efficacy and toxicity of anti-tumor agents because they cannot mimic the complexity of the organism. The generation of 3D models with the integration of various cell types, novel matrix materials and mechanic effects allows a better understanding of tumor physiology and drug effects than the conventional 2D cultures.

In this review, the spectrum of different 3D models in BC research is illustrated by listing which cells and matrices were successfully used to study specific aspects of BC development and metastasis. Based on the different subtypes of BC, the role of the components of the tumor microenvironment (TME) is summarized in the first part, and the general composition and application of 3D BC models in the second part of the text. A detailed description of the individual models and production techniques is beyond the aim of this review.

## 2. Types of Breast Cancer and Metastasis

BC originates from the lactiferous ducts, which are embedded in a stroma composed of fibroblasts, adipocytes, blood and lymphatic vessels, and immune cells. Ductal carcinoma in situ (DICS), which is limited to the epithelium and does not penetrate the basal membrane, can be classified into the same molecular subtypes as invasive BC using gene expression analysis. The tumors differ in their hormone receptor status; luminal-A-like has estrogen receptor (ER)+/progesterone receptor (PR)+/human epidermal growth factor receptor 2 (HER2)− expression, luminal-B-like ER+/PR−/HER2− and HER2-enriched ER+/PR−/HER2+. Triple-negative tumors lack ER, PR and HER2 expression. The ER is highly expressed in approximately 70–75% of invasive BCs. The PR is expressed in more than 50% of ER-positive tumors and rarely in ER-negative BCs. The proliferation marker Ki-67 is low in luminal A, variable in luminal B, and high in HER2+-enriched BCs and TNBCs [7]. The prevalence of the BC types varies between the studies and was reported between 31–86% for luminal A type, 6–16% luminal B, 2–16% HER2-enriched and 4–26% for triple-negative (TNBC or basal-like) tumors [8]. The BC subtype was associated with the tumor prognosis; HER2-enriched had 7.5% local and 3.4% regional recurrence and TNBC tumors had 7.6% local and 3.3% regional recurrence. Luminal A subtype tumors were recurrent in 1.5% and 0.7% local and regional, respectively, and the luminal B subtype was associated in 2.9% and 1.5% of the cases with local and regional relapse [9]. In addition to the receptor type, invasive BCs are subjected to the TNM classification. Roughly, this classification starts with the tumor diameter of 2 cm as T1, and tumors with either chest or skin invasion are classified as T4 [10]. Lymph node categories start at 1–3 axillary nodes as N1 and N3 as >10 axillary or infraclavicular lymph nodes. At M1, distant metastases including contralateral lymph nodes were identified. The parameters can be determined either by pathology or by imaging techniques, and the categories also include the “0” state. However, M0 is not a valid category because a biopsy of a suspicious lesion does not guarantee the absence of pathology.

**Figure 1:**
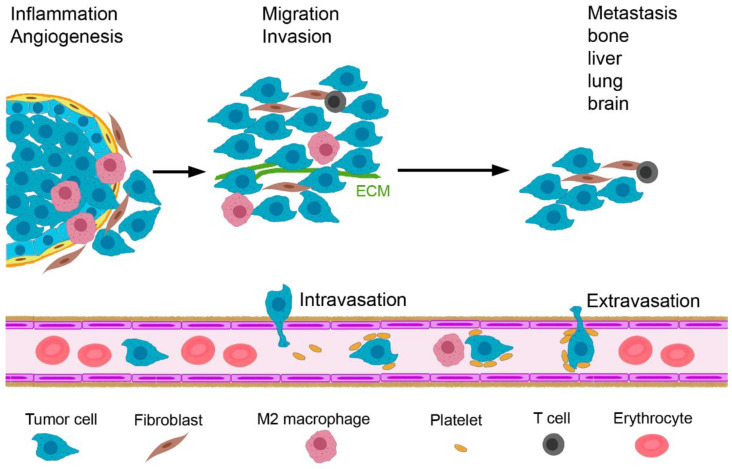
Important steps in the metastasis of invasive breast cancer. Tumor initiation is favored by inflammation, angiogenesis and hypoxia. Upon tumor progression, tumor cells migrate, invade the stroma and intravasate into the blood vessels. Tumor cells in the circulation are protected by platelets and activate monocytes to M2 macrophages. Extravasation takes place supported by macrophages and neutrophilic granulocytes and guided by a cytokine gradient, and metastasis in bone, liver, lung and brain occur. Abbreviation: ECM, extracellular matrix.

Invasive BC starts when tumor cells proliferating in the lumen of the mammary alveoli breach the barrier formed by myoepithelial cells and the basement membrane (Figure 1). By a combination of genetic changes that enable the tumor cells to invade the adjacent tissue, and an abnormal microenvironment that leads to disruption of the basement membrane, tumor cells spread into the stroma [11]. Subsequently, metastasis occurs as a sequential process, comprising invasion and migration, intravasation, extravasation and growth at the metastatic site. Inflammation, hypoxia and angiogenesis occur during the initiation of tumor growth. Under the influence of transforming growth factor β (TGF-β), the BC cells perform epithelial–mesenchymal transition (EMT) and invade and migrate locally. Subsequently, they attach to the endothelial wall and intravasate. BC cells circulate in the blood as single cells or as multi-cellular aggregates together with platelets that protect them against apoptosis and shear stress [12]. Supported by M2 macrophages and neutrophilic granulocytes, they can exit the blood vessels (extravasation) [13,14]. At the arrival site, they are dormant in order to adapt to the new environment.

## 3. Tumor Microenvironment

The evaluation of tumor cells without their TME is artificial because there is an intense interaction between tumor and stromal cells. Stephen Paget was the first to identify the important role of the TME in tumorigenesis and tumor progression. Paget’s main interest was in the formation of metastases, and he postulated the seed and soil theory for their formation [15]. In the seventies and eighties of the 20th century, researchers realized the importance of blood vessels, macrophages, fibroblasts and immune cells for tumor progression [16]. The TME is characterized by a low extracellular pH and a high level of hypoxia, similar to chronic wounds; it stimulates tumor cell growth and survival and attracts other cells into the tumor by the secretion of growth factors and chemokines [17]. The extracellular matrix (ECM) provides physical support but becomes disorganized in later tumor stages. The amount of stroma has a different role in the BC types. For ER+ tumors, increased stroma content is related to improved outcome, in TNBCs with poor survival [18].

**Figure 2:**
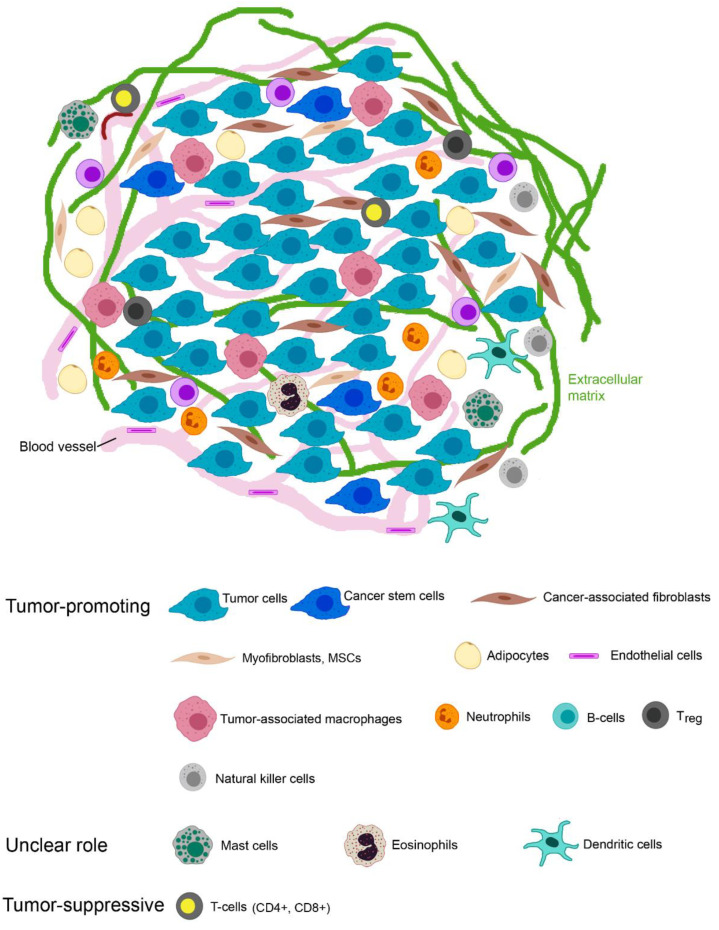
Breast cancer tumors consist not only of tumor cells and cancer stem cells but contain other cells with tumor-promoting functions such as cancer-associated fibroblasts, normal fibroblasts, myofibroblasts, mesenchymal stem cells, tumor-associated adipocytes, endothelial cells and various immune cells (tumor-associated macrophages, neutrophils, natural killer cells and regulatory T-cells). The role of mast cells, eosinophils and dendritic cells in breast cancer is not clear. CD8+ cytotoxic T-cells and CD4+ helper T-cells have anti-tumor action.

Cancer stem cells (CSCs) characterized by CD44+CD24^LOW-NEG^ and aldehyde dehydrogenase (ALDH1) activity, possess self-renewal, a high proliferation rate and the generation of a heterogenic lineage [19]. By the overexpression of ATP-binding cassette (ABC) transporters, increased ALDH1 activity, increased DNA repair and elevated reactive-oxygen-species (ROS)-scavenging capacity, CSCs are resistant to anti-tumor treatment [20]. The cells of the TME include cancer-associated fibroblasts (CAFs), tumor-associated adipocytes (TAAs), endothelial cells, and immune cells, particularly tumor-associated macrophages (TAMs) (Figure 2). Dendritic cells (DCs), natural killer (NKs) cells, mast cells (MCs), granulocytes, myeloid-derived suppressor cells (MDSC), and B- and T-lymphocytes play an important role in the balance between tumor progression and tumor control. T-lymphocytes comprise memory T-cells, cytotoxic CD8+ T-cells (CTL) and different subsets of CD4+ T helper (Th) and immunosuppressive regulatory T-cells (Tregs). To underline the need to include them in the 3D culture, their role is briefly described in the next sections.

### 3.1. Cancer-Associated Fibroblasts

Fibroblasts are physiologically active only upon the involution of the mammary gland after weaning. In this phase, they express α-smooth-muscle actin (α-SMA) and are termed myofibroblasts. CAFs are regarded as activated myofibroblasts in cancer [21] and can represent up to 80% of the BC mass [22]. Based on their immunocytochemical staining against fibroblast activation protein α (FAP), fibroblast-specific protein 1 (FSP), vimentin, α-SMA, integrin β1/CD29, platelet-derived growth factor (PDGF) receptor β and caveolin 1 (CAV1), they are subdivided into four populations (S1–S4). CAF-S2 and CAF-S3 appear like normal fibroblasts. Luminal A and B tumors are characterized by the predominance of CAF-S2, whereas TNBCs have a high presence of CAF-S1 and CAF-S4, which are seen only in cancer [23]. CAF-S1 is defined by ECM remodeling, adhesion, wound-healing and immunosuppressive properties, whereas CAF-S4 is characterized by contraction and a metastasis-promoting signature [24].

CAFs mainly originate from fibroblasts, but origination from BC cells, CSCs, endothelial cells, mesenchymal stem cells (MSCs), adipose tissue MSCs and pericytes is also possible. It was also found that CAFs, similar to tumor cells, circulate in the body [25,26]. The hypothesis is that they help circulating BC cells to populate new areas [27]. CAFs secrete various growth factors, such as epidermal growth factor (EGF), TGF-β, PDGF, vascular endothelial growth factor (VEGF), insulin growth factor (IGF), and hepatic growth factor (HGF) [28]. In addition, proteins and ligands of the tumor necrosis factor (TNF) pathway, the chemo- and cytokine stromal-cell-derived factor 1 (SDF-1), motif chemokine ligand (CXCL)-14, CXCL-16, chemokine ligand (CCL)-5, interleukin (IL)-4, IL-6 and IL-17A are secreted. Matrix metalloproteinases (MMPs) and their inhibitors and components of the ECM (collagens, fibronectin, laminin, hyaluronic acid) are further important secretion products. Together with hypoxia inducible factor (HIF)-1α for metabolic reprogramming, these proteins are key players in the effects of CAFs and cause the induction of the EMT, promotion of proliferation, survival and migration of BC cells, self-renewal of CSC, angiogenesis, immunosuppression, ECM production and remodeling, and metabolic reprogramming of BC cells. The immunosuppressive effect by CAFs is achieved by the polarization of macrophages and neutrophils to the immunosuppressive phenotype and the inhibition of NK cell and DC action [29]. CAFs induce TH1 to TH2 polarization, inhibit CD8+ and stimulate Treg differentiation, and foster the recruitment and differentiation of MDSCs. They are also involved in resistance against trastuzumab [30]. MSCs have tumor-promoting effects when exposed to chemical (TGF-β) and mechanical (stiff ECM) stimuli [31]. They are recruited to the TME and act either directly or after differentiation to CAFs on the BC cells. They can stimulate angiogenesis, the EMT of BC cells, increase stemness, induce drug resistance and modulate immune competence [32]. However, anti-tumor action (e.g., induction of apoptosis and sensitization to radiotherapy) has also been reported [33]. More information on the origin, phenotype and role of CAFs can be found elsewhere (e.g., [34,35]).

### 3.2. Tumor-Associated Adipocytes

TAAs differ from normal adipocytes by their reduced lipid content. They support tumor growth by the release of high-energy metabolites (pyruvate, ketone bodies, free fatty acids), produce collagen and release pro-inflammatory cytokines and MMPs [36]. Adiponectin is the only anti-tumor cytokine, whereas the other cytokines secreted by TAAs, which include IL-6, TNF, VEGF and IL-1β, promote the induction of CAFs and fibroblast-like endothelial phenotypes. TAAs have overlapping roles with CAFs and promote angiogenesis, tumor cell survival and proliferation, maintain cancer stem cells and recruit adipocytes and macrophages to the tumor site [14]. TAAs also confer resistance against chemotherapy and radiotherapy. The secretion of versican, which favors vascularization and promotes angiogenesis, is typical of TAAs. Adipocyte-derived stromal cells are present in normal breast adipose tissue and have the potential to differentiate into various cell types of the mesenchymal lineage. The differentiation depends on the ECM composition and sex hormone levels. The inability to differentiate into adipocytes and osteoblasts is linked to invasive BC growth in obese women. Chronic inflammation was postulated as a potential reason for this loss. Adipocyte-conditioned media increased the migration of MCF-7 cells, thereby promoting metastasis [37].

### 3.3. Endothelial Cells

Endothelial cells of blood vessels line the leaky vasculature tumor network; they trigger angiogenesis and modulate immune responses [14]. Blood vessels lead to a better supply of the tumor cells with oxygen and nutrients combined with a better clearance of waste products. Further, the contact of BC cells with vessels increases the mesenchymal characteristics of the tumor cells and promotes a more aggressive phenotype. The tumor-associated endothelial cells have a higher expression of the stem cell marker ALDH1, upregulated VEGF and VEGF-receptor 1 + 2 levels and increased activities of MMP-2 and MMP-9. The high VEGF levels lead to a disordered arrangement of the leaky vessels. Endothelial cells of lymphatic vessels supported the tumor growth of MDA-MB-231 cells in xenografts by PDGF-induced pericyte infiltration and angiogenesis [38]. TNBCs, which contain increased VEGF-C and VEGF-D levels, are characterized by dense lymphatic networks and increased aggressiveness and metastasis.

### 3.4. Immune Cells

A variety of immune cells can be found in the tumor stroma. The most important group is the tumor-associated myeloid cells, which include TAMs, myeloid-derived suppressor cells (MDSCs), tumor-associated neutrophils, Tie2-expressing monocytes (paracrine inducers of angiogenesis and tumor growth with Tek tyrosine kinase receptor expression) and tumor-associated DCs [39]. TAMs represent the most abundant cell type and may represent >50% of the cells of the TME. TAMs and MDSCs are distinct cell types but not clearly distinguished because they share common characteristics. Immune cells, particularly TAMs, are good examples to illustrate the dynamic changes in the TME. Macrophages can be recruited to the tumor site during early oncogenesis as pro-inflammatory M1 cells [40]. In this initial stage, they act as anti-tumorigenic and destroy tumor cells. After prolonged activity, chronic inflammation and genetic instability promote tumor proliferation and progression. In parallel, BC cells reprogram TAMs to the M2 phenotype with tumor-promoting properties.

It is generally assumed that only M2-like TAMs are responsible for tumor progression, but M1-like TAMs may also contribute because they sustain inflammation and cause angiogenesis by the secretion of IL-1β, TNF-α, and IL-6 [41]. In this review, the expression of TAMs refers, if not indicated otherwise, to the M2-like TAMs, which display their tumor-promoting effects by immunosuppression, angiogenesis, and metastasis. The immuno-modulating action is due to IL-10 secretion and altered arginine metabolism to decrease the T-cell response and lower MHC class II expression needed for antigen presentation [39]. Further, TAMs stimulate angiogenesis by VEGF and HIF-2α secretion, remodel the ECM by urokinase-type plasminogen activator and type I collagen production, and support cancer stemness through IL-6 secretion and EGF/EGF receptor signaling. Tumor promotion occurs through the alteration of metabolism by providing the tumor cells with polyamines, ROS, lactic acid, Lipocalin and heme oxygenase (HO)-1. TAMs further increase invasion and metastasis and treatment resistance. MDSCs act via similar mechanisms on immune suppression and support cancer stemness, tumor invasion and metastasis.

Tumor-associated neutrophils are able to stimulate the EMT, sustain the survival of TAMs and promote the drug resistance of BC cells [14]. They further facilitate angiogenesis, mutagenesis and suppression of the immune system [42]. By forming neutrophil extracellular traps, they can shield the tumor from the immune system [40]. Tregs (CD8+FoxP3+) disrupt the immune control in BCs. TH2 CD4+ cells promote BC progression and metastasis by stimulating EGF signaling and favoring the M2 polarization of macrophages [43].

Mast cells can suppress the immune response and may have pro- and anti-cancer effects in BC [44]. DCs are involved in anti-tumor defense and targets of anti-cancer vaccines. In BC, they are often dysfunctional/poorly activated and their role is unclear [45]. There are also unclear results on the role of eosinophiles; they can have anti-tumoral or pro-tumoral functions, according to activation signals in the TME [46]. Innate lymphoid cells have three subclasses (IDC1–IDC3). Although they usually have anti-tumor action, together with stromal cells, they may promote metastasis in BC. However, their role is still a matter of debate [47].

Infiltrating CD8+ cytotoxic T-cells and CD4+ helper T-cells, by contrast, have anti-tumor action, and there is a good correlation of their amount to good prognosis in TNBC [43]. However, any correlation of these immune cells to prognosis is missing in luminal A tumors.

### 3.5. Myoepithelial Cells

Myoepithelial cells (MECs) morphologically resemble smooth-muscle cells but express specific cytokeratin filaments typical of epithelial cells [48]. These cells form the outer layer of the mammary gland epithelium and transport milk to the nipple by their contraction. They could prevent propagation of the tumor by presenting a barrier because only 50% of ductal carcinoma in situ progress to invasive BC. MECs can suppress tumor progression by inducing breast cell polarization and producing anti-angiogenic factors and proteinase inhibitors. Consequently, BCs with a defective MEC layer have poor prognosis [49]. In some BC cases, however, MECs promote cancer growth by the secretion of CXCL14 and CXCL12 chemokines.

### 3.6. Extracellular Matrix

The ECM of BC resembles the matrix upon the involution of the mammary gland and has a subtype-specific composition. The mammary gland is subjected to cyclic restructuring of the ECM, which makes the tissue more vulnerable than other organs to dysregulation [50]. Luminal-type BCs have less stiff ECM with less immune cell infiltration than TNBCs, where collagen linearization and deposition leads to increased stiffness and increased infiltration by immune cells. Main components include the proteins collagen I, III and V, elastin, vitronectin, the glycoproteins laminin-111, laminin-332 and fibronectin, and the proteoglycans hyaluronan and chrondroitin sulfate [51]. The increased stiffness is due to the increase in the fibrillary collagens (I, III, V), fibronectin and hyaluronan [52]. The increased matrix proteins further include tenascin C, periostin, osteopontin, thrombospondin 1 and SPARC (secreted protein, acidic and rich in cysteine). The high levels of MMPs produce active metabolites, such as the MMP-2-induced degradation of laminin-332, which results in the production of a degradation product with EGF-like activity.

Interaction between BC and stroma cells in the TME occurs via the secretome, which includes proteins (growth factors, cytokines, enzymes), extracellular vesicles containing proteins, and microRNA. It is hypothesized that BC cells transform adipocyte-derived MSCs into myofibroblast-like cells and that these cells secrete VEGF, SDF-1 and TGF-β to increase angiogenesis [40]. The secretome also contains proteases, particularly the plasminogen-plasmin system and the matrix metalloproteinases for the remodeling of the ECM [53].

## 4. Models

The aim of in vitro cancer models is to mimic BC in vivo for a better understanding of tumor initiation and metastasis (invasion and migration, intravasation and extravasation of tumors cells and potential growth at the most common sites of bone, liver, lung and brain; Figure 1). The main applications of the models are drug efficacy testing, the identification of biomarkers and new tumor targets, the characterization of drug resistance mechanisms and the selection of drugs for personalized treatment. The elucidation of the role of the cellular components of the TME and of the stiffness and porosity of the ECM in these processes are major research themes that require physiologically relevant BC models.

The compound screening of anti-tumor agents is routinely performed in 2D cultures of cancer cell lines. The most often used cell line to mimic luminal A BCs (ER+/PR+/HER2−) is the Michigan Cancer Foundation (MCF)-7 line. The T47D line belongs to the same subtype, whereas BT474 and ZR75.1 are luminal B BCs, and SKBR3 and UACC-893 are examples of HER2+ BCs [54]. The cell lines MDA-MB-231 and the MDA-MB-468 from the Monroe Dunaway Anderson (MDA) Cancer Center, BT20, HCC38, HCC1937, HCC1806, SUM149, SUM159 and SUM1315 cells represent TNBCs. The conventional culture system allows for fast screening but lacks the spatial arrangement and composition of various cell types, the modulation of ECM properties, relevant shear stress and hydrodynamic pressure values, and continuous supply with nutrients/removal of waste.

The easiest method to generate 3D cultures from these cells is preventing them from adhering to the culture vessel. Three BC cell lines in monoculture were assessed in hanging drops, liquid overlay and suspension culture and the following differences observed [55]. MCF-7 cells formed spheroids under all conditions, MDA-MB-231 only under one liquid overlay condition, and SKBR3 under no conditions. Co-culture with other cells changed the natural propensity of the tumor cells to form spheroids, e.g., fibroblasts improved the ability of MDA-MB-231 cells to form spheroids [56].

### 4.1. More Relevant Culture Conditions: Inclusion of More Cell Types

To improve the physiological relevance of the models, co-cultures of BC cells with other cell types were performed. Either co-culture in one vessel allowing physical contact or culture of one cell type and transfer of conditioned media from (an)other cell(s) was used. The direct co-culture of the cells is rarely possible for more than two cell types over prolonged times because medium requirements are different. Suboptimal co-culture conditions may favor the growth of one cell type and decrease the viability of other cells. The different optimal growth conditions also limit the use of tumor explants, which include all relevant cells at the start of the culture but change in composition over time. A good way to maintain the differentiation and survival of stromal and immune cells is the culture of tissue pieces embedded in collagen on the membrane of a transwell system [57]. In this technique, termed air–liquid interface culture, the apical compartment of the transwell contains air and only the basal side growth medium. This culture is also suitable for 3D models.

In co-culture models for shorter time periods (24–72 h), combinations of cells can be seeded. Ten thousand MDA-MB-231 cells were seeded with 500–5000 RAW264.7 macrophages and good spheroid formation were observed only in the presence of 5000 macrophages [58]. Yakavets et al. reported the importance of the seeding ratio on the formation of spheroids [59]. They generated spheroids from MCF-7 and MRC-5 cells in ratios varying from 2:1 to 1:5 and found that the fibroblasts differentiated into myofibroblasts. When the ratio of MCF-7:MRC-5 exceeded 2:1, the fibroblasts clustered in the center and were not distributed across the spheroid. In this situation, direct interaction between tumor cells and fibroblasts appears unlikely. These examples show that methodological issues influence the cellular composition of the BC spheroids and that the ratio between the cell types may differ from the situation in vivo. The tumor mass of BCs may consist of up to 80% CAFs, but fibroblasts could not be integrated into spheroids to >33% in the MCF-7/MRC-5 co-culture model. Cell ratios also differ between the models. In a complex microfluidic model of a vascularized bone-mimicking microenvironment, cells were seeded in final concentrations of 0.75 × 10 × 10^6^/mL MDA-MB-231, 0.1 × 10 × 10^6^/mL primary fibroblasts, 4 × 10 × 10^6^/mL HUVECs, and 0.1 × 10 × 10^6^/mL each MSCs and MSC-differentiated osteocytes [60]. In another, less complex microfluidic model, 16 × 10 × 10^6^/mL MDA-MB-231 and 0.5 × 10 × 10^6^/mL fibroblasts were seeded [61]. For the extrusion printing of BC models, 1–2 × 10 × 10^6^/mL MDA-MB-231 and 0.8–1.25 × 10 × 10^6^/mL NIH/3T3 fibroblasts in 3% alginate solution were printed [62]. In layered models, the cancer cell layer contained 3 × 10 × 10^6^/mL MDA-MB-231 cells and the vascular layer 5 × 10 × 10^6^/mL HUVECs plus 1 × 10 × 10^6^/mL lung fibroblasts in 1% alginate/0.15% collagen I [63]. These examples show that cell numbers and matrix compositions are variable and complicate comparisons between models.

For direct co-culture, the cell culture medium has to be adapted in such a way that not only the BC cells but also the stromal and immune cells survive [64]. Variations in medium composition can be pronounced because even the simple Minimal Essential Medium (MEM) contains thirteen amino acids, eight vitamins, six ionic species and glucose [65]. Further, the commonly used supplement fetal bovine serum (FBS) is very complex and has variable quality. To enable co-culture, either the mixture of media in different ratios or a basal media with different supplements is possible.

As an alternative to direct co-culture, conditioned media may be used. The secretome, which comprises secreted substances produced by tumor cells, stromal cells, endothelial cells, and immune cells within the TME, has key importance for tumor propagation and metastasis [66]. The addition of the media from other cells (conditioned media) to cancer cells is therefore a possibility for indirect co-culture. Conditioned medium from differentiated adipocytes reduced the in vitro sensitivity of BT474 and SKBR3 to the tyrosine kinase inhibitor lapatinib [67]. When working with conditioned media, the selection of the optimal amount of the conditioned medium is challenging because, on the one hand, sufficient nutrients and stimulants should be present and, on the other, the level of waste products should not be too high [65]. Particularly, the concentrations of ammonium (cytotoxic) and lactic acid (lowering of pH) is critical.

### 4.2. More Relevant Culture Conditions: Inclusion of ECM

The presence and composition of the ECM is an important component of realistic models. Although spheroids produce their own ECM, the embedding in natural or synthetic matrices can reveal the importance of chemical and mechanical stimuli in tumor physiology. Matrigel, commercialized for instance as Cultrex™ or Geltrex™, is rich in laminin (60%), collagen IV (30%), entactin/nidogen, heparin sulfate and growth factors and, despite some negative aspects, is the most often used matrix.

In addition to Matrigel, collagen and agarose were used for the embedding of spheroids [68]. Agarose has the limitation that cancer cells are not able to remodel the matrix. In a study with MDA-MB-231 cells, collagen I was chosen as the matrix because its strength, pore size and fibril fraction can be varied. The mechanical properties of 0.25% to 0.8% collagen I as the embedding medium were superior to Matrigel. Higher concentrations of Matrigel (2.5–5%) improved the spheroid formation of BC cells [69]. The embedding of spheroids in collagen I provides the possibility to study the migratory population at the periphery of the spheroids and compare their properties to the non-migratory, CSC-containing core of the spheroid. The prominent effect of the matrix stiffness was demonstrated when pre-adipocytes and BC cells were cultured in gelatin methacrylate (GelMA) hydrogel with tunable stiffness, and the HCC1806 and MDA-MB-231 cells inhibited the differentiation into adipocytes only at high stiffness values [70]. The importance of the ECM was also seen for bioprinted MCF-7, MDA-MB-231, T47D and SKBR3 cells, which contained a higher proportion of CSCs when cultured on fibrous polycaprolactone (PCL) scaffolds than in plastic culture dishes [71]. It is not clear whether only the 3D environment or also the mechanic properties of the scaffold caused these differences.

Biobased scaffolds can be produced from gelatin, gelatin/alginate and collagen/alginate for the combination of MCF10A, MCF-7, MDA-MB-231 or patient-derived BC cells and adipose-derived MSCs [72]. Gelatin is widely used because it needs no functionalization and can easily be generated from denatured collagen. The surface of alginate is usually covalently modified with adhesion peptides (arginine–glycine–aspartic acid = RGD) to improve the interaction with the cells. As an alternative, combinations of alginate with Matrigel or gelatin can be used. A comparison of MCF10A, MCF-7 and MDA-MB-231 cells in 2% Matrigel, 1% sodium alginate/10% gelatin and 3% alginate/0.025% collagen I showed that all inks worked equally well and all BC cells and HUVEC showed high viability [73]. MDA-MB-231 cells were viable when bioprinted in 4% gelatin/4% alginate hydrogel [74]. PEG hydrogel generated from a four-arm poly(ethylene glycol) maleimide and bis-thiol activator enabled the monitoring of the growth and migration of MCF-7 and MDA-MB-231 cells, suggesting that synthetic hydrogels are suitable for the bioprinting of BC cells [75]. Collagen I concentrations in microfluidic channels were in the range of 0.1% [61].

De-cellularized breast tissue proved to be very promising as a scaffold for bioprinting [76]. The production was reproducible and comparable to Matrigel and collagen matrices. Even when using this matrix, from the physiological standpoint perspective, the structure of the re-constructed matrix was different from the one in vivo. De-cellularized and de-lipidated breast tissue as the physiologically most relevant growth substrate needs to be modified for bioprinting. After combination with GelMA and alginate, it was a suitable bio-ink for the printing of MCF-7 and human-adipose-tissue-derived MSCs to produce BC models [77]. Since the bone microenvironment confers drug resistance in BC cells, a bone matrix was studied as the scaffold [78]. It has been shown that nanocrystalline hydroxyapatite (nHAp) particles promoted the adsorption of serum proteins and enhanced BC cell growth. Hydroxyapatite is a ceramic material, and it is challenging to fabricate 3D in-vitro models from them directly. Therefore, combinations such as polyethylene glycol (PEG)/hydroxyapatite (HAp) matrix for the co-culture of fetal osteoblasts + MDA-MB-231 and PCL/tricalcium phosphate for MDA-MB-231, SUM1315 and osteoblasts were used. Another option was the use of osteoblasts or MSCs and MDA-MB-231 printed in GelMA/HAp matrix [79]. The data showed that all matrices supported BC cell growth.

Polymer scaffolds, e.g., PEG, polyvinyl alcohol (PVA), PCL, polylactide-co-glycolide (PLG) were more rarely used than natural matrices, although mechanical properties can be better tuned with synthetic than with natural scaffolds, and the functionalization of a PEG–heparin hydrogel with collagen and laminin increased the biocompatibility [80].

### 4.3. Engineered 3D Tissues

Reconstructed tissues were developed in skin research and represent stratified layers of cells. BC models were formed on porous gelatin microspheres. Dermal fibroblasts, HUVECs and finally MCF-10 or MCF-7 cells were seeded on the beads and angiogenesis was determined according to the diameter of the branching vessels [81]. The layering of CAFs and MCF-7 cells on these microspheres was used for drug screening [82]. The layering on microspheres could present an alternative to the encapsulation of MCF-7 or MDA-MB-231 cells in biosynthetic (PEG diacrylate conjugated to natural fibrinogen) hydrogel [83].

### 4.4. Spheroids/Organoids

**Figure 3:**
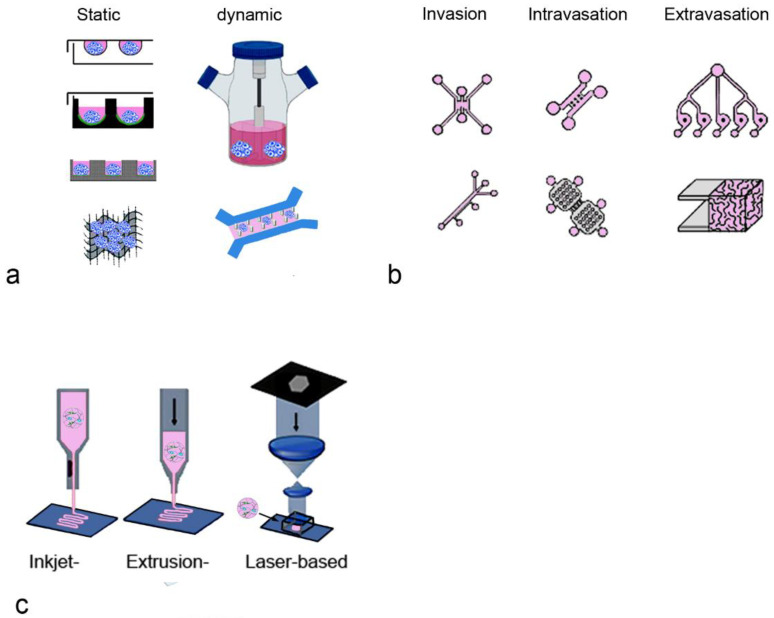
Examples of the production of BC models are shown. (**a**) Static (hanging drop, ultra-low attachment, micropatterned plates, scaffolds) and dynamic methods (spinner flask, microfluidic system) to generate spheroids [86]; (**b**) microfluidic systems with different geometries depending on the studied process [89]; (**c**) models produced by various printing techniques [90].

Spheroids were the first 3D cancer cell models. The most common techniques include the static, scaffold-free methods of hanging drop, liquid overlay on ultra-low attachment plates, and micropatterned plates [84]. There are also dynamic methods such as the spinner flask, rotating vessels, microfluidic systems or techniques using scaffolds for the generation of spheroids (Figure 3a). Detailed descriptions of the available techniques can be found elsewhere [85,86].

Mainly BC cell lines were used for their generation because the cell lines are more available and easier to culture than primary cells. However, the success in generating cell lines is lower (15%) for BCs than for colorectal, lung and thyroid cancers (≥25%) [87]. BC tissue contains a high percentage of fat cells and a relatively low volume of epithelial cells. Further, primary BC cells compared to other primary tumors (e.g., colon or prostate), are difficult to isolate from other cell populations. Therefore, available BC cell lines were mainly derived from metastases or pleural effusions, which leads to the situation that the less-aggressive subtypes are poorly represented [88].

**Figure 4:**
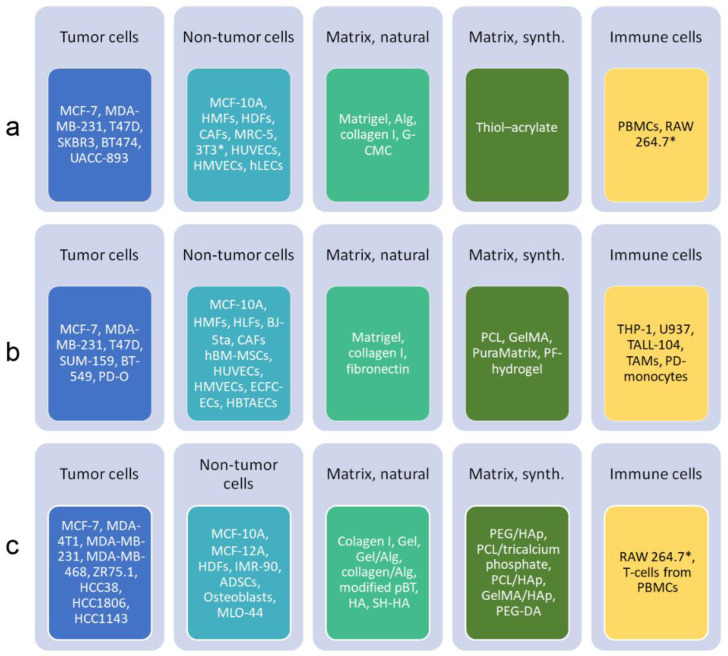
Overview of cells and scaffold materials, which were used in spheroids or organoids ((**a**), [104]), microfluidic systems ((**b**), [50,105]) and 3D bioprinting ((**c**), [106,107]) in BC research. The focus is on TME and bone metastasis. Due to lack of space, cells used in models of metastasis to other organs are not included. Abbreviations: ADSCs, adipose-derived stem cells; Alg, alginate; BJ-5ta, hTERT-immortalized fibroblasts; CAFs, cancer-associated fibroblasts; ECFC-ECs, endothelial colony-forming cells; G-CMC, gelatin-carboxymethyl cellulose; Gel, gelatin; GelMA, gelatin methacrylate; HA, hyaluronic acid; HAp, hydroxyapatite; HBTAECs, primary human-breast-tumor-associated endothelial cells; hBM-MSCs, human bone marrow mesenchymal stem cells; HDFs, human dermal fibroblasts; hLECs, human lymphatic endothelial cells; HLFs, human lung fibroblasts; HMFs, human mammary fibroblasts; HMVECs, human microvascular endothelial cells; HMT1937, non-tumorigenic breast cells; HUVECs, human umbilical vein endothelial cells; IMR90, fibroblasts; MLO-44, osteocyte-like cells; PBMCs, peripheral blood mononuclear cells; pBT, porcine breast tissue; PCL, polycaprolactone; PD, patient-derived; PEG, polyethylene glycol; PEG–DA, polyethylene glycol diacrylate; PF, polyethylene glycol/fibrinogen; RAW264.7, macrophages; SH-HA, hyaluronic acid with thiol groups; 3T3, fibroblasts; TALL-104, T-lymphoblast cell line; TAMs, tumor-associated macrophages; THP-1, monocytic leukemia cells; U937, pro-monocytic myeloid leukemia cells. Murine cells are marked by asterisk.

Spheroids consisting of BC cells alone or in combination with other cell types have been generated and examples of the cells and materials are shown in Figure 4a to illustrate which cells or combinations of cells formed spheroids with BC properties. They were designed to address specific research questions. It is not possible to decide which 3D model better reflects the physiology of BC in patients because the authors compared their models to 2D cultures or tumors in vivo and not to other 3D models.

Tumor cell heterogeneity in spheroids can be mimicked if the cells are already included at the spheroid formation. To this end, breast cells with no (MCF-10A cells), low (MCF-7 cells) and high (BT20 cells) invasive potential were co-cultured [91]. It is assumed that, using this model, the reaction of BC cells with different invasive and metastatic potential can be better studied.

The effect of the TME was mimicked by the inclusion of stromal cells. The co-culture of UACC-893, BT20 or MDA-MB-453 with foreskin fibroblasts showed immunocytochemical characteristics of BCs [92]. With the exception of the study by Angelucci et al., CAFs and normal fibroblasts had pro-tumorigenic effects [93]. In combinations of BT474, T47D, MCF-7 or SKBR3 cells with CAFs or fibroblasts and MDA-MB-231 or MCF-7 with CAFs or skin fibroblasts, the invasive properties of the BC cells and drug resistance were increased [59,94,95,96,97]. Similar results were obtained when spheroids were generated in alginate matrices [98,99]. Murine 3T3 and human fibroblasts showed similar effects [100]. These data suggest that (i) fibroblasts without prior contact to tumor cells can promote tumor development, (ii) interspecies differences play no role in the effect, and (iii) the presence of alginate does not affect the tumor-promoting effect.

Tevis et al. also showed that for the polarization of macrophages, murine and human cells react similarly [58]. They observed the polarization of murine RAW264.7 macrophages into the M2 phenotype when embedded together with MDA-MB-231 cells in collagen. The model, therefore, may be suitable to study the effect of TAMs on drug responses. For personalized immunotherapy, peripheral blood mononuclear cells (PBMCs) from BC patients should be used because it was shown in mixed spheroids with MDA-MB-231 that the immune cells exhibit a wide range of anti-tumor responses and allogenic PBMCs with HLA-mismatch could lead to incorrect results [101]. The degradation of the ECM was increased when MDA-MB-231 cells were co-cultured with HUVECs in collagen gel [102]. HUVECs increased the proliferation and size of spheroids in another study [103].

The overview (Figure 4a) shows that mainly established BC lines were used for the generation of spheroids. Fibroblasts from different sources and either PBMCs or murine macrophages were included as components of the TME. For the formation of the spheroids or as the embedding media, a small spectrum of natural matrices was used.

In contrast to spheroids, organoids, which can be obtained from different tumor stages, can represent intratumoral heterogeneity due to the self-renewal capacity of the CSCs. BC cell lines (e.g., MCF-10A, MCF-7, SUM149, SUM159, SUM1315 and MDA-MB-231) also contain CSCs but they are not suitable for personalized treatment [108].

Organoids generated from patient tumors are mainly used for personalized treatment. They are further used for genomic and biological studiesfor instance, to study the effect of progesterone and estrogen in BRCA1-mutated BC [109]. They serve as a source for xenografts, as models for immunotherapy and for biobanking. Protocols have improved over the years, but organoid production is still more costly and less standardized than that of spheroids. Limitations of their routine use in personalized treatment include the variation of the success rate to generate organoids with the BC type, the unclear influence of the available ECM materials and the use of only small tumor parts, which may not be representative of the entire tumor. Organoids could be generated from >90% of BCs, which was similar to the generation of spheroids from patients’ cells, (>90%), when co-cultures with normal fibroblasts were performed. The majority of the organoids showed the same immunocytochemical staining pattern as the original tumor [110]. Similarly, the immunocytochemical characterization (ER, PR, HER2, transcription factor GATA-3) of the cancer cells in the spheroids corresponded to that of the primary tumor. The authors also showed that the reaction to drugs was similar to that observed in xenografts and patient responses. The high success rate of organoid generation from BC, however, decreased to 65% if, instead of treatment-naïve primary tumors, organoids from metastatic sites of drug-treated patients were generated [111].

Since the organoids represent only cells of epithelial origin, protocols for the co-culture with CAFs and immune cells (e.g., macrophages) were developed [112,113]. Similar to findings in spheroids from BC cell lines, CAFs increased the invasive behavior of BC cells in patient-derived organoids [114]. The interaction of T-cells with BC cells was studied by the co-culture of BC cells with blood-derived γδ T-cells over 2–3 weeks [115]. The co-culture was obtained either by the addition of immune cells to the BC organoids or by the dissociation of the entire tumor tissue and subsequent co-culture. Co-culture of patient-derived organoids with immune cells was seen as more problematic than for cell lines when the allogenic immune cells were HLA-mismatched. Air–liquid interface culture was estimated as essential to prolong survival of the immune cells. Colorectal cancer organoids with CD45+ cells could be cultured for 10 days but the amount of CD3+ cells decreased [116]. The maintenance of NK, T- and B-cells and macrophages in the cultures for several months was also reported.

There are currently no standard protocols for the generation of organoids, which is a major limitation because it was reported that culture conditions affect the success rate more than the features of primary tumors, including the clinical stage, treatment conditions before organoid generation, and pathological diagnosis [117].

### 4.5. Microfluidic BC Models

Microfluidic systems can include additional physiological parameters and have been used in various settings (Figure 3b). They have the advantage that the platforms can also be used for biopsy material [118]. After dissociation with collagenase, the suspension can be applied with collagen in the central channel and cell culture medium in the adjacent channels on both sides to form the 3D model. The low requirement for cells enables the culture of biopsies in 8 days, which is sufficient for drug screening [119].

Some studies incorporated handing drop, cell-laden agarose or alginate microgels and microwell-based arrays in the system, but the microfluidic platform itself can also be used to generate spheroids. By the encapsulation of tumor cells into a large number of Matrigel-in-oil droplets and the subsequent removal of the oil, uniform tumor spheroids were formed within a few hours via Matrigel-supported cell self-assembly [120]. Another possibility for spheroid production is the use of microwells connected by a common channel that supplies them with cell culture medium [121]. The spheroids in the well consisted of tumor cells and ECM-mimicking hydrogel (gelatin and cellulose nanocrystals). Size and shape were visualized by fluorescently labeled microgel. The authors reported very homogenous sizes and shapes of MCF-7 spheroids and low variations in the response to doxorubicin using this system, making this system ideal for drug screening.

The great advantage of the chip technology compared to the static well system consists of the more realistic extracellular space to cell relation, the continuous medium supply and the possibility to assess the effect of shear stress. The relevance of flow was shown by using high and low perfusion. When MCF-7 and MDA-MB-231 formed colonies with elongated (high perfusion rate) or round dormant (low perfusion) morphology. The same change in morphology was also observed in CAFs [122] and reflects the situation of cells with different morphologies in tumor tissue in vivo. By the variation of the perfusion of MCF-7 and MDA-MB-231 in fibrinogen hydrogel, it turned out that doxorubicin and paclitaxel were much more efficient in high-perfusion than in low-perfusion conditions [122]. The different drug efficacy depending on tumor perfusion may be relevant for drug selection and enable personalized treatment.

Microfluidic systems proved to be suitable to mimic the specific situation of DCIS, where tumor cells have to grow in a lumen formed of normal mammary cells. To mimic this situation, MCF-10 cells were seeded in a lumen formed by a hydrogel containing fibroblasts [123]. After the MCF-10 cells had formed a monolayer, MCF-10-DCIS cells were seeded inside the lumen. MCF-10-DCIS (initially called MCF-10-DCIS.com) is a clonal BC cell line derived from a xenograft originating from premalignant MCF-10AT cells that were injected into immune-deficient mice. In an alternative model, MCF-10-DCIS spheroids were cultured in a two-channel microfluidic system. The spheroids were cultured on a layer of MCF-10 cells and an ECM membrane separated the apical channel from the basal channel, which contained fibroblasts in collagen gel [124].

The steps of tumor initiation and metastasis, namely invasion into the ECM, intravasation into the blood vessels, extravasation from the blood vessels and metastatic growth, were studied in various microfluidic systems (Figure 4b). The small medium volumes and channel depths allowed cell tracking by microscopy. A panel of cell lines was seeded into a feeder of parallel Y-shaped collagen-coated channels [125]. The relative migration and proliferation of the cells correlated well with the metastatic potential of the cells. The model was refined in such a way that endothelial cells, CAFs or immune cells were included. MDA-MB-231 migrated into collagen gels to greater extent in the presence of HUVECs [126]. The cell morphology, expression profile and migration of SUM159 cells were more similar to aggressive tumors in co-culture with CAFs than with fibroblasts [113]. In this setting, the BC cells and fibroblasts or CAFs in Matrigel/collagen hydrogel were injected into different channels of a microfluidic device. Other models showed the tumor-promoting effect of monocytes. The migration of BC cells in a co-culture model of T47D or MDA-MB-231 with monocytes in a collagen matrix of variable stiffness was enhanced because the monocytes differentiated into TAM [127]. The density of the 3D collagen matrix and stimulation with macrophage colony-stimulating factor (M-CSF) were identified as important factors in the regulation of BC migration. Intravasation into lymphatic vessels has not been studied by microfluidic systems so far. There is a need to develop such systems because the process is of key importance in early-stage cancer [128].

The passive intravasation through a leaky endothelium can be simulated with conventional transwell assays, where cells are seeded on a porous membrane. The main improvement by microfluidic systems consists of the fact that the fluid stress can be varied. When active intravasation is studied, there has to be a direct interaction of tumor cells with endothelial cells to enable the disruption of the endothelial monolayer [129]. The situation was studied in different settings to assess the influence of the TME. Using a model containing MDA-MB-231, collagen (stromal compartment) and HUVECs in fibrin (vascular compartment) in three independent but interconnected compartments, Nagaraju et al. found that the endothelial cells stimulated intravasation into the vascular compartment and increased invasion into the stromal compartment [130]. By the combination of MDA-MB-231 with MSCs, it was found that co-culture increased growth and sprouting of the spheroids in the 1:1 (MDA-MB-231: MSC) mixture but not when cells were mixed in a 1:3 ratio [131]. Further, the sprouting was higher in Matrigel than in the Matrigel/collagen I matrix. The dependence of the observed effect on the ratio of the seeded cell types was also reported in a static spheroid model [59]. This suggests that similar effects are visible in static and dynamic culture systems. The role of PBMCs was evaluated in a model where BT474 cells were seeded alone or together with CAFs, PBMCs, or CAFs + PBMCs in the lateral chambers of a device. The central chamber contained a HUVEC monolayer [132]. The presence of CAFs increased the invasive potential of the BC cells, while PBMCs were important for the action of trastuzumab. The monoclonal anti-HER2 antibody increased cancer–immune interactions and induced an anti-tumoral antibody-dependent cell-medicated cytotoxicity (ADCC) immune response in the presence of PBMCs. Some microfluidic models exploited the self-assembling property of endothelial cells to generate interconnected vessels inside the lateral channels. Spheroids containing MCF-7 and normal fibroblasts in a central channel and HUVECs in two lateral channels of the microfluidic device evaluated the propensity of the BC cells to migrate into the endothelium [133]. Further, various other combinations of endothelial cells and fibroblasts embedded in hydrogels together with tumor cells have been published [50]. The assembled vessels were perfused with monocytes and the effect on the invasion of MDA-MB-231 from the adjacent channel was observed [134]. The model was further refined in such a way that MDA-MB-231 were present in one channel and TAM with endothelial cells in the other channel [135].

For extravasation, tumor cells were added into endothelial-cell-coated channels to provide the tumor cells direct access to endothelial cell receptors. Song et al. (2018) constructed a chip where the HUVEC-coated channel contained either non-tumorigenic (MCF-10A) or tumorigenic (MCF-7, MDA-MB-231) cells. The BC cells were surrounded on both sides by a channel with medium and more peripherally by a channel containing human lung fibroblasts [136]. With such models, the potentially relevant influences (e.g., hypoxia or inflammation) could be assessed. A system consisting of three fibrin-filled parallel channels separated by two medium channels was used to include the effect of blood cells in extravasation [137]. HUVECs were seeded in the central channel and primary lung fibroblasts in the two other channels to promote vessel formation. A mixture of platelets, neutrophils and MDA-MB-231 cells was injected in one of the medium channels. The authors showed that the presence of platelets and neutrophils increased extravasation.

Independent from the type of BC, bone is the most frequent site of metastasis. Prevalence is highest in the hormone-receptor-positive/HER2− tumors at 58.52% and lowest in the triple-negative BCs at 36.39% [138]. Therefore, most researchers have concentrated on this metastasis model. In a co-culture system consisting of HUVECs with bone-marrow-derived MSCs and MDA-MB-231 on decellularized bone tissue in a microfluidic system, the endothelial cells formed a network and supported BC cell survival without the external addition of growth factors [139]. Another model consisted of MDA-MB-231 inside a 3D microfluidic lumen lined with HUVECs adjacent to a channel seeded with osteocyte-like MLO-44 cells. The osteocytes were mechanically stimulated, which resulted in reduced extravasation of the tumor cells [140]. To identify preferential metastatic sites, circulating tumor cells were pumped through HUVEC-lined channels that separated this compartment from others containing primary cells from lung, liver, muscle and bone [141]. The higher cell number in the liver than in the bone and muscle channels correlated to a higher liver metastasis in mouse xenografts. MDA-MB-231 cells also survived in the presence of hepatocytes and not further identified non-parenchymal cells in PEG scaffolds functionalized with cell-adhesive peptide [142]. In a very complex 16-unit microchannel network, the extravasation of MDA-MB-231 cells into different compartment containing A549, BEL7402 and U87 was conducted to simulate metastasis into lung, brain and liver [143].

Microfluidic systems are very flexible regarding the use of the cells or biopsies, but the size of the tumor tissue, which can be cultured in microfluidic systems (10 × 10^6^ cells compared to 10 × 10^9^ in vivo) may be limited [144]. The use requires more equipment and technical knowledge than the generation of spheroids. Despite the advantages of better control of the culture conditions by the inclusion of sensors for pH, temperature, oxygen concentration and fluid flow in the chip, the limited technical robustness (e.g., presence of air bubbles) requires expert knowledge.

The panel of cells used in microfluidic techniques (Figure 4b) resembles the one listed for spheroid models. A greater variety of fibroblasts and endothelial cells included in the models was reported, while the panel of natural and synthetic matrices was similar to the spheroid models.

### 4.6. 3D Bioprinting

As mentioned in Section 3.2. a wide range of matrices support the growth of BC cells. However, the interaction of tumor cells and stromal cells is expected to be influenced by the properties of the ECM. For a systematic evaluation of the influence of the ECM, bioprinting is suitable because this technique can use any biocompatible natural or synthetic polymer matrix that favors adhesion and cell growth [145] (Figure 3c). For the generation of 3D models in wells and microfluidic systems, a limited number of mainly natural matrices is used. Due to their good availability, they also dominate as materials in bioprinting. Lobo et al. summarized the existing literature on bioprinting in BC and concluded that natural materials such as alginate, collagen, and derivatives were used more often (81.82%) than synthetic ones (18.18%) [107]. Natural biomaterials can be isolated from tissues and cells and include collagen, fibrin, alginate and chitosan. Their disadvantage is that only one compound of the ECM is present. The use of Matrigel circumvents this problem but the composition is not constant. The de-cellularized ECM contains the complete mixture of components of the ECM and is a good scaffold for bioprinting. The synthetic polymers can be functionalized with peptideRGD) or fibrinogen, collagen type I or laminin-111 to promote adsorption and cell adhesion. Apart from biocompatibility issues, the formation of spheroids in the printed matrices depends on the intrinsic properties of the cells. When injected in a linear manner within rat collagen, rat mammary ECM, and human mammary ECM, MCF-7 cells formed round structures, whereas MDA-MB-468 tissues only formed small spheroids when grown in rat mammary ECM [76]. This result is surprising because not the allogenic but the xenogenic material was the preferred substrate.

Bioprinted tumor models can be generated by extrusion, inkjet, stereolithography-based bioprinting, laser-assisted and electrospinning-based bioprinting. The main 3D-printing technologies employed are extrusion (61.11%) and laser printing (16.67%) [107]. The respective techniques will not be described in this review, and the reader is referred to reviews dedicated to this topic, e.g., [106,146,147].

Similar cell types as for spheroid generation and microfluidic technologies are also used for bioprinting (Figure 4c).

Many bioprinted models include only BC cells. MCF-7 or MDA-MB-231 in rat collagen I were studied [73]. MDA-MB-231, MDA-MB-468, MCF-7 and SKBR3 cells were printed in GelMA and assessed for sensitivity to cytostatic agents [148,149]. Patient-derived BC cells were bioprinted in PEG hydrogels, functionalized with adhesion peptides and in gelatin-derived hydrogels (GelMA and crosslinked thiolated gelatin). Organoids were formed in these gels and reacted with doxorubicin and paclitaxel at higher concentrations than in 2D cultures [150].

Mixed spheroids of MDA-MB-231 and IMR-90 fibroblasts in alginate/gelatin hydrogel were formed in such a way that the MDA-MB-231 cells aggregated to spheroids and later the IMR-90 infiltrated the spheroids [151]. Mixed spheroids were also generated by the printing of MCF-7, HCC1143, SKBR3 and MDA-MB-231 cells into a stromal mix of human mammary fibroblasts (HMFs) and HUVECs in alginate and gelatin [152]. Depending on the origin of these cell lines, the spheroids had different morphologies. No data about the invasive potential of the mixed spheroids compared to spheroids consisting only of tumor cells were presented and, therefore, the effect of the co-culture on the tumor phenotype is unknown.

Several bioprinting studies focused on the role of MSCs in BC for a better understanding of bone metastases. Adipose-derived MSCs enhanced the migration of fluorescently labeled MDA-MB-231 cells in a laminin and collagen IV matrix [153]. Adipose-derived MSCs increased the resistance to doxorubicin of primary BC cells bioprinted in methacrylated hyaluronic acid/gelatin hydrogel [72]. Further, the presence of the MSCs increased growth and migration of MDA-MB-231 cells in GelMA hydrogel with nanocrystalline hydroxyapatite (nHAp) [154]. MDA-MB-231 cells with fetal osteoblasts printed in PEG/PEG–diacrylate/nHAp matrix were used for the study of bone metastasis [155]. The BC cells co-cultured with osteoblasts within the 3D bone matrix formed multi-cellular spheroids. The tumoroid formation of MCF-7 or MDA-MB-231 cells was also observed in a PCL/HAp clay scaffold [142]. It was also shown that MDA-MB-231 cells reduced the activity of alkaline phosphatase, a differentiation marker for osteocytes, in a co-culture of bone-marrow-derived MCSs or osteoblasts when cultured together in GelMA/nHAp [156]. MSCs or osteoblasts, by contrast, increased the proliferation of BC cells. These studies suggest that both adipocytes and osteocytes promote the growth of BC cells.

The overview (Figure 4c) of the cells used in bioprinting lists more BC cell lines and a greater variety of cell types included in the models than in BC spheroids. The majority of the screened articles (75.86%) used MDA-MB-231 and MCF-7 cells [107]. Natural and synthetic matrices are much more varied than those used in the other techniques. While more complex matrices can be produced by 3D bioprinting, the high-throughput and the easier assessment of interactions with vasculature are advantages of microfluidic systems [157].

There is also the possibility to combine the techniques. One example is the study of T-cell migration using spheroid formation, 3D photopatterning and microfluidics [158]. Spheroids of MCF-7 or MDA-MB-231 cells formed in Matrigel and THP-1 monocytes were encapsulated within the inner layer of GelMA hydrogel, while HUVECs were loaded in the outer layer of a microfluidic channel. When the constructs were perfused with media containing T-cells (TALL-104), cultures with BC spheroids recruited significantly more T-cells compared to groups with dispersed cells. Moreover, the presence of monocytes synergistically increased T-cell recruitment into the tumor site.

## 5. Selection of the Appropriate Model

The selection of the appropriate model depends on the research question and is dictated by the availability and knowledge of the technique, time and costs. The advantages of spheroids/organoids were described as easy to integrate multiple cell types, uniform size of the engineered tissues and the possibility of use for high-throughput screening [159]. The advantages of cancer on a chip were listed as follows: fine control of the microenvironment, good mass transport and the ability to integrate sensors [160]. The advantages of bioprinting are a complex architecture, spatial control, precise reproducibility and low costs [161]. A summary of advantages, limitations and preferential use of the 3D BC models is presented in Table 1.

The best model would be the least complex one based on the available technologies, but able to provide meaningful results. Therefore, for the first screening of compounds in drug development, 3D systems might not be needed and the standardized proliferation, invasion and migration assays with a panel of BC cell lines may be sufficient. For proliferation, viability assays based on dehydrogenase activity (MTT and similar assays) are commonly used. The invasion and migration of cancer cells is routinely studied with simple, well-standardized assays, the permeation of matrix-coated transwell membranes for invasion (spheroid invasion) and wound healing assay, the tracking of cell motion or permeation of transwell membranes for migration, or the angiogenesis assay. These assays can be refined by using co-cultures with cells from the TME to assess their influence on the invasive potential. Depending on the experimental condition, the promotion and inhibition of MDA-MB-231 migration have been published [162,163].

For drug testing in personalized treatment, patient-derived organoids, ideally in co-culture with CAFs and/or immune cells, are requested. Monocultures of patient-derived organoid cultures may provide sufficient information on the sensitivity of the tumor cells. Studies on drug resistance require the presence of stromal cells because CAFs, MSCs, TAAs and neutrophils support this effect. It may, however, be sufficient to include only CAFs in the model to obtain information about the effect of the TME. The inclusion of macrophages could be relevant for the testing of anti-cancer vaccines and macrophage-targeted nanocarriers [164]. It is, however, not clear how the tumor–stroma interaction models can be applied to personalized therapy for the patients. It was, for instance, shown that matrices derived from murine tissue (e.g., Matrigel) activate CD4+ cells due to the presence of antigens in the gel [165].

The lack of expert knowledge in specific technologies represents a hindrance to using more refined models. Platforms for the generation of spheroids are commercially available and flexible regarding the number of included cell types. To give one example: MCF-7 or MDA-MB-231 plus HUVECs and human lung fibroblast spheroids were generated by In-Sphero technology and vascularization studied in a microfluidic device [166]. Experimental conditions for correct testing have been published, because it was found that organoids can very well predict drug sensitivity in vivo in patients and mice provided that viability of the generated organoids is sufficiently high [167].

If researchers do not have the needed expertise, the equipment or the cell source for the generation of 3D cultures, spheroids are commercially available. The InSight™ microtissues by InSphero are composed of cancer cells of different origin (cell lines, patient-derived cells) or of co-culture of cancer cells with CAFs or fibroblast cell lines. Biobanks can provide organoids for BC, colorectal, prostate lung cancer and glioblastoma [168]. Further, several commercial platforms provide organoids using different patented platforms, mainly based on hydrogel technology. Some examples are Known Medicine, Cypre, and Grown Bioscience.

There are also (usually microfluidic-based) commercial platforms for the generation of 3D models from cancer tissues. Major suppliers are Mimetas, Synvivo, 4Design Biosciences and AIM Biotech. The microfluidic OrganoPlate^®^ platform has been used to study the effect of paclitaxel, olaparib and cisplatin on MDA-MB-453, MDA-MB-231 and HCC1937cells [169]. It may be expected that commercially available platforms and tissues will lead to a greater standardization of the testing and increased use in drug screening. An overview of companies selling cancers on a chip is provided at https://www.elveflow.com/microfluidic-reviews/organs-on-chip-3d-cell-culture/organ-chip-companies/ (accessed on 21 December 2022).

Commercial solutions for bioprinting presently focus on bio-inks and the production of organs for regenerative medicine. However, Ioannidis et al. reported that they were able to convert a 3D printer into an open-source 3D bioprinter and provide a customized bio-ink based on accessible open-source alginate/gelatin precursors [170]. Such technical solutions make the technology easier to use and lead to a more reproducible generation of BC models.

## 6. Analysis Tools of 3D Model

Optical detection is the most relevant technique for the characterization of the 3D models (e.g., shape and size of the spheroids/organoids, cell composition) and visualization of invasion and migration. In microfluidic systems, cellular motion can be monitored by conventional brightfield or fluorescence microscopy. An analysis of the structures inside the 3D models requires advanced microscopic techniques because light scattering, light absorption and autofluorescence hinder the penetration and detection of fluorescent signals. Spheroids are mostly imaged after fixation and clearing with one of the several available protocols [171]. The authors did not report major differences between the methods TDE, RapiClear, Opticlear and CUBIC. Two-photon microscopy or light sheet microscopy are the most common microscopes used for the imaging of whole spheroids. Alternatively, 3D tissues can be fixed, embedded in paraffin or cryomatrix, sectioned and stained. Sections of embedded 3D models can be stained with histologic staining (Hematoxylin/eosin for cells, Masson’s trichrome for collagen deposition). Immunocytochemistry and other fluorescent staining (DAPI, Hoechst, phalloidin, live/dead) is also possible. Imaging of live spheroids/organoids is more complicated because clearing methods cannot be applied and the excitation of fluorescent dyes damage the cells.

**Figure 5:**
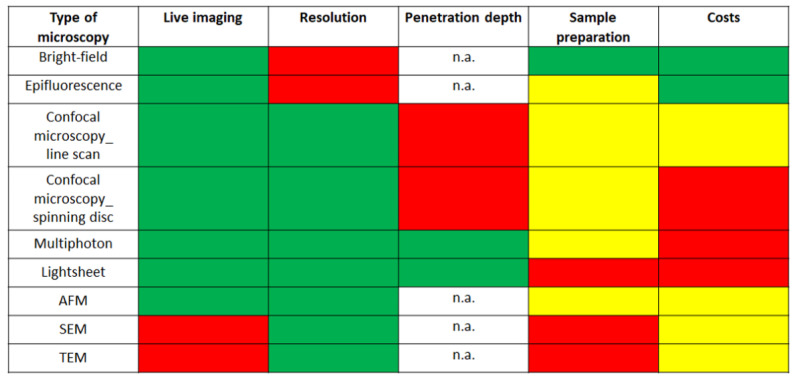
Evaluation of the imaging techniques based on their use in live imaging, resolution, penetration depth, sample preparation and costs. Green color indicates a high, yellow an intermediate and red a low score. n.a., not applicable.

For the identification of surface structures, scanning electron microscopy (fixed tissue) or atomic force microscopy (fixed and live samples) may be used. The advantages and disadvantages of several microscopic techniques are shown in Figure 5 [172].

Other tools for the analysis of 3D models include colorimetric and spectroscopic techniques (for cytotoxicity and cytokine release), flow cytometry, Western blot and qRT-PCR, [173]. Spectrometric assays (MTT, MTS, Alarmar blue, Celltiter-Glo 3D) are useful to assess cell metabolism and cell viability [146]. Cytokines in the supernatant/perfusate are routinely quantified by colorimetric assays. Flow cytometry enables the single-cell analysis of cell cycle, apoptosis and marker expression. Western blot and RT-qPCR are often used techniques, whereas RNAsec and DNA methylation arrays and proteomic techniques are more rarely employed [174].

A histological analysis of the tissues is complicated by their small size, whereas for flow cytometry and single-cell analysis, the appropriate dissociation techniques have to be selected because prolonged enzymatic treatment leads to cell damage.

## 7. Conclusions

The currently available 3D BC models represent many aspects of tumor physiology much better than the previously used 2D cultures. Specific characteristics of the TME, namely the tumor-promoting properties of CAFs, endothelial cells, MSCs and monocytes, can be mimicked by 3D models with all technologies. The fact that the number and relative composition of different cell types included in the models, chemical and mechanical properties of the matrices, and the presence of shear forces markedly influence the reaction of the 3D models complicates the interpretation and comparison of the obtained data between studies. The available techniques for the generation of BC models have different strengths and limitations as summarized in Table 1. For the generation of spheroids, no specific equipment is needed and various cell types can be integrated. Organoids are currently the best option for personalized treatment, although they represent only a small part of the tumor, and the efficacy of organoid generation from the samples is below 100%, which means that not all patients can profit from this method. Microfluidic systems are very versatile tools and can also be used for biopsies because the amount of cells is low. They allow control of the environment and the inclusion of sensors and enable continuous monitoring. The influence of the properties of the tumor matrix and spatial relationships can be best studied in bioprinted models because they can provide high cellular complexity, variable matrix architecture and spatial control. Limitations of the models include lack of growth control, reduced micro-architectural controllability and the missing possibility of the long-term culture of spheroids and organoids [175]. High costs, non-physiological geometries, and a lack of comparison between different settings were listed as limitations of microfluidic systems [175]. The inclusion of blood vessels, poor biocompatibility, the lack of bioactive ligands and the presence of degradation products may pose problems in bioprinting [176].

The general problems with the existing 3D models are that mainly cell lines are used, which has the disadvantage that inter- and intratumoral heterogeneity is poorly represented. The use of FBS as a supplement in the media and Matrigel as a scaffold limits the reproducibility and standardization of the models. Compared to other cancers, TAAs play an important role in BC and should be contained in the TME. Their integration, however, is problematic.

## Figures and Tables

**Table 1:** Overview of advantages, disadvantages and main applications of 3D BC models.

Technique	Advantage	Disadvantage	Application
Spheroids	Easy and standardized production, inclusion of many cells	Little control of formation	Cellular interactions, angiogenesis, drug testing
Organoids	High similarity to primary tumor	Not representative for entire tumor, not possible for all tumors	Cellular interactions, drug screening for personalized medicine
Microfluidic systems	Spatial and temporal control, low requirement of cells, integration of sensors	Problems with flow (shear stress, air bubbles), complex geometry, lack of ECM	Cellular interactions, angiogenesis, chemotaxis, intravasation/extravasation
3D printing—natural matrices	High biocompatibility, high cell adhesion, modulation by cells possible	Batch variability, uncontrolled degradation	Role of stiffness, migration, invasion, angiogenesis, hypoxia
3D printing—synthetic matrices	Highly tunable	Poor cell adhesion, lack of biocompatibility	Role of stiffness, migration, invasion, angiogenesis, hypoxia
3D printing—composite material	Biophysical and biochemical properties can be adjusted	Very complex, cytotoxicity, batch to batch variation	Role of stiffness and porosity, migration, invasion, angiogenesis, hypoxia
Decellularized scaffolds	Properties and composition similar to in vivo	Changes in properties by the treatment, residual treatment agents	Cellular interactions

## Data Availability

No new data were created or analyzed in this study. Data sharing is not applicable to this article.
